# Prognostic Value of Adrenomedullin and Natriuretic Peptides in Uroseptic Patients Induced by Ureteroscopy

**DOI:** 10.1155/2016/9743198

**Published:** 2016-01-05

**Authors:** Wei Hu, Pang-hu Zhou, Wei Wang, Lijun Zhang, Xiao-bin Zhang

**Affiliations:** ^1^Department of Urology, Renmin Hospital of Wuhan University, No. 238 Liberation Road, Wuhan, Hubei 430060, China; ^2^Department of Orthopedics, Renmin Hospital of Wuhan University, No. 238 Liberation Road, Wuhan, Hubei 430060, China

## Abstract

The aim of this paper is to investigate whether urosepsis is related to irrigation pressure of ureteroscopy (URS) and evaluate the prognostic value of adrenomedullin (ADM) and atrial and brain natriuretic peptides (ANP and BNP) in URS-induced uroseptic patients. From July 2008 to October 2013, we enrolled 332 patients with untreated unilateral ureteral obstruction (UUO). The UUO group included three subgroups of, respectively, 118, 132, and 82 patients who underwent URS under intermittent stable irrigation pressure of, respectively, 80, 120, and 160 mmHg. The plasma concentrations of ADM, ANP, and BNP were measured in all subjects. URS was performed for all UUO patients; the values of the three peptides were measured again after URS. Irrigation pressure and stone size were independent risk factors of urosepsis. After URS, the plasma concentrations of ADM, ANP, and BNP were significantly higher in uroseptic patients. Moreover, the concentrations were significantly higher depending on the disease severity. Plasma concentrations of the three peptides were correlated with plasma ET concentration in the uroseptic patients. The areas under receiver operating characteristic (ROC) curve of ADM, ANP, and BNP for predicting urosepsis were 0.811, 0.728, and 0.764, respectively. In conclusion, ADM, along with ANP and BNP, is valuable for prognosis in urosepsis secondary to URS which is associated with irrigation pressure.

## 1. Introduction

Urosepsis is defined as sepsis caused by infection of the urogenital tract and is a systemic response to infection [1]. Frequent causes for urosepsis are obstructive diseases of the urinary tract, such as ureteral stones, anomalies, stenosis, or tumor [2]. It can also occur after interventions in the urogenital tract, such as transrectal prostate biopsy, percutaneous nephrolithotomy (PCNL), or ureteroscopy (URS) [3–5]. Early diagnosis and timely intervention have great importance for urosepsis. Effective treatment in the early periods of urosepsis can prevent irreversible organ damage and reduce mortality. Therefore, finding useful biomarkers plays a crucial role in early diagnosis of urosepsis.

Adrenomedullin (ADM) was first isolated from human pheochromocytoma [6]. It has been detected in the plasma and other fluids of normal individuals [7]. Adrenomedullin possesses anti-inflammatory, bactericidal, positive inotropic, and, perhaps most importantly, vasodilatory activities [8]. Several clinical studies have demonstrated that ADM increases significantly in septic patients and is correlated with disease severity, which is valuable for prognosis in septic patients [9, 10].

Atrial and brain natriuretic peptides (ANP and BNP, resp.) are polypeptide hormones comprising the cardiac-derived natriuretic peptide system which are involved in the long-term regulation of sodium and water balance, blood volume, and arterial pressure [11–13]. Increased plasma ANP and BNP have been identified as predictors of cardiac dysfunction in sepsis and prognosis in patients with congestive heart failure or ischemic heart disease [14, 15]. Many reports indicate that ANP or BNP levels are elevated in septic patients and they can provide useful diagnostic and prognostic information in septic patients [14, 16, 17].

To measure the plasma concentrations of ADM, ANP, and BNP and evaluate their prognostic value in uroseptic patients induced by URS, we measured the values of the three peptides in untreated patients with unilateral ureteral obstruction (UUO) secondary to ureteral stones and compared the results with those of healthy control subjects. Additionally, we measured the values of the three peptides after URS for UUO patients.

## 2. Materials and Methods

### 2.1. Study Subjects

This retrospective, case-control study was conducted in Renmin Hospital of Wuhan University between July 2008 and October 2013 in accordance with our institutional standards and under the appropriate license of the Ethics Committee of Renmin Hospital, as well as in adherence to national regulations. The study groups consisted of 90 healthy control subjects (50 men and 40 women, mean age 41.3 ± 12.6 years, range 18 to 66 years) and 332 patients with untreated UUO (185 men and 147 women, mean age 43.3 ± 12.0 years, range 18 to 68 years). In order to investigate whether urosepsis is related to irrigation pressure of ureteroscopy (URS), all UUO patients underwent URS with intermittent stable irrigation pressure of 80 mmHg (group I, 66 men and 52 women, mean age 43.2 ± 12.1 years; range 18 to 68 years), 120 mmHg (group II, 73 men and 59 women, mean age 43.3 ± 11.6 years; range 19 to 66 years), and 160 mmHg (group III, 46 men and 36 women, mean age 43.6 ± 12.8 years; range 19 to 67 years), respectively. All patients agreed to participate in this study and provided written informed consent. The operation was performed by only one experienced surgeon. The irrigation pressure was randomly selected and the patients and surgeon were blind to it according to the double blind method. All patient information was anonymized and deidentified prior to analysis. Routine laboratory studies and image examinations were conducted before URS. The inclusion criteria for UUO subjects were as follows: simple unilateral ureteral stone, without urinary tract infections by urinalysis (the presence of 10 urine leukocytes/HPF and no microorganisms), without any symptoms of urogenital tract infections, without antimicrobial prophylaxis before the procedure. Ureteroscopy was performed using a Wolf rigid ureteroscope. UUO was definitely diagnosed on the basis of intravenous pyelonephrography or computed tomography. All patients undergoing laser lithotripsy had a double-J stent and a Foley catheter placed at the end of the procedure. The indwelling Foley catheter was drawn within 72 hours. All uroseptic patients were symptomatic or signs of potential sepsis were present within 24 hours after URS. They fulfilled the systemic inflammatory response syndrome (SIRS) criteria defined by the American College of Chest Physicians/Society of Critical Care Medicine [18], regardless of the procedure duration and residual stones. Patients who developed sepsis were treated with vasopressors such as phenylephrine and norepinephrine to sustain blood pressure when necessary. None of these UUO patients had clinical evidence of active infection, malignant cancer of any type, acquired immunodeficiency syndrome (AIDS), end-stage renal or liver disease, diabetes, pulmonary disease, valvular heart disease, congenital heart disease, acute myocarditis, angina pectoris, myocardial infarction, essential hypertension, or other diseases. Control subjects were age- and gender-matched healthy subjects who had been hospitalized for a health checkup.

### 2.2. Assay Procedures

Venous blood samples were drawn from an antecubital vein within 24 hours for nonseptic subjects or septic patients before vasopressor treatment and were transferred to ice-chilled tubes containing Trasylol (500 KIU/mL) and ethylenediaminetetraacetic acid (EDTA, 1 g/L). They were then centrifuged at 3,000 rpm for 15 min at 4°C and the plasma was immediately frozen and stored in polypropylene tubes at −80°C until radioimmunoassay (RIA).

Baseline clinical data were recorded as follows: endotoxin (ET) using the Pyrochrome test kit (Pyroquant Diagnostik GmbH, Mörfelden, Germany), white blood cell counts (WBC) using a Sysmex SE-9000 analyzer (Toa Medical Instruments, Kobe, Japan), C-reactive protein (CRP) using an immunoturbidimetric assay (Modular Analytics P, Roche Diagnostics, Mannheim, Germany), lactate (LAC) using enzymatic method (Modular Analytics P, Roche Diagnostics, Mannheim, Germany), and procalcitonin (PCT) using an immunoassay analyzer (Block Scientific, Bohemia, NY).

The plasma ADM levels were measured with specific RIA for human ADM (ADM RIA SHIONOGI, Shionogi Pharmaceutical, Co., Ltd., Osaka, Japan). The intra- and interassay coefficients of variation were 3.8% to 7.9% and 4.5% to 8.8%, respectively. All assays were performed in duplicate. ADM concentrations were expressed as ng/L.

The plasma ANP concentrations were measured with a specific immunoradiometric assay for human ANP (Shiono RIA ANP kit, Shionogi and Co., Osaka, Japan). The intra- and interassay coefficients of variation were 4.7% to 9.8% and 5.9% to 11.6%, respectively. The plasma BNP concentrations were measured by a method similar to that for ANP, developed by the same company (Shiono RIA BNP kit). The intra- and interassay coefficients of variation were 5.8% to 10.7% and 6.5% to 12.5%, respectively. All assays were performed in duplicate. The concentrations of ANP and BNP were expressed as ng/L.

### 2.3. Statistical Analysis

All continuous data were expressed as mean ± SD and analyzed with SPSS software, version 19.0 (SPSS Inc., Chicago, IL). Comparisons between two variables were performed with unpaired *t*-test or Mann-Whitney *U* test. Multiple comparisons were evaluated with analysis of variance followed by Student-Newman-Keuls or Kruskal-Wallis method. The significance of differences between paired variables was determined by paired *t*-test or Wilcoxon test. Categorical variables were assessed by the chi-square test or Fisher's exact test. Stepwise multiple linear regression analysis was used to evaluate the most important factor for ADM, ANP, and BNP. The correlation between two variables was done by linear regression analysis and further confirmed by Spearman's rank test. Receiver operating characteristic (ROC) curves were used to predict urosepsis and determine the cutoff values. Kaplan-Meier curves were plotted according to the identified cutoff values of ADM, ANP, and BNP and further confirmed by log-rank test. A 2-sided *p* value less than 0.05 was considered to indicate statistical significance.

## 3. Results

Table 1 shows the baseline characteristics of the study groups. There were no significant differences in age, sex distribution, WBC, and plasma concentrations of ET, CRP, LAC, PCT, Scr, and Ccr among the four groups. No significant differences were observed in stone side distribution, stone site, and stone size among the three UUO subgroups.

After URS, the uroseptic rates of the three UUO subgroups were 8.5% (10/118), 18.2% (24/132), and 30.5% (25/82), respectively, which were significantly higher and higher in proportion to the irrigation pressure (*p* < 0.05). However, there were no significant differences in age, sex distribution, Scr, and Ccr among the three uroseptic groups (data not shown).

We analyzed the risk factors of urosepsis by stepwise multiple logistic regression analysis which revealed that stone size (*B* = 0.695, OR = 2.004, *p* = 0.024) and irrigation pressure (*B* = 0.750, OR = 2.118, *p* = 0.000) were the most important independent factors of urosepsis, when age, sex, stone side, stone site, stone size, and irrigation pressure were taken into account.

Table 2 shows the clinical parameters at diagnosis and after URS in uroseptic patients. As expected, WBC and plasma concentrations of ET, CRP, LAC, and PCT were significantly higher after URS than at diagnosis (*p* < 0.05). However, Scr and Ccr remained unchanged after URS.

**Figure 1:**
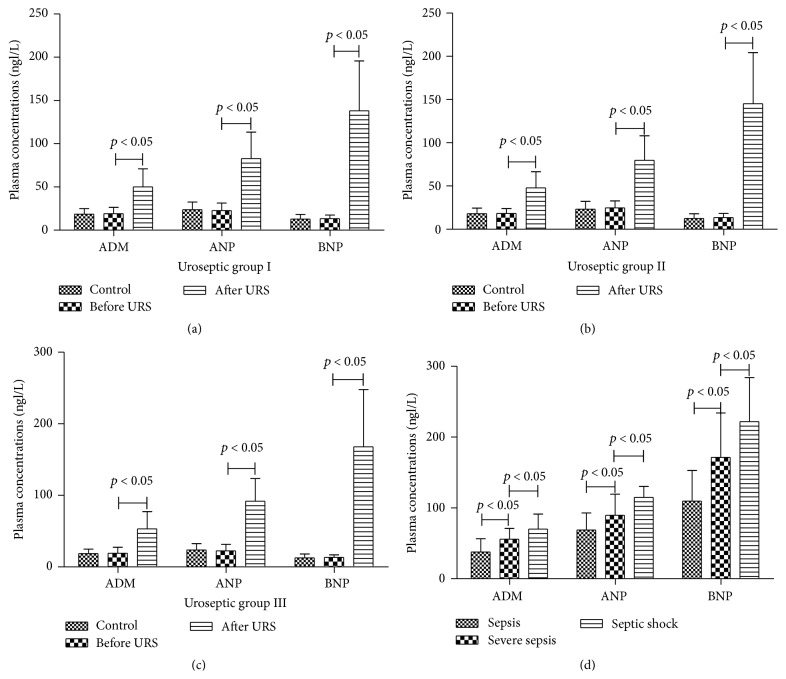
(a) Plasma concentrations of ADM, ANP, and BNP in controls and uroseptic group I before and after URS under 80 mmHg. (b) Plasma concentrations of ADM, ANP, and BNP in controls and uroseptic group II before and after URS under 120 mmHg. (c) Plasma concentrations of ADM, ANP, and BNP in controls and uroseptic group III before and after URS under 160 mmHg. (d) Plasma concentrations of ADM, ANP, and BNP in URS-induced sepsis, severe sepsis, and septic shock.

The plasma concentrations of ADM, ANP, and BNP in controls and uroseptic patients before and after URS are depicted in Figures 1(a)–1(c). The mean value of ADM was significantly higher in uroseptic group I after URS (50.19 ± 20.67 ng/L) than before URS (19.08 ± 7.36 ng/L) and in controls (18.50 ± 6.46 ng/L) (*p* < 0.05). There was no significant difference in mean ADM value between uroseptic group I before URS and controls. The mean values of ANP in controls and uroseptic group I before and after URS were 23.63 ± 8.98, 22.56 ± 8.70, and 82.91 ± 30.43 ng/L, respectively, while the mean values of BNP were 12.72 ± 5.52, 13.24 ± 4.11, and 137.97 ± 57.79 ng/L, respectively. Similar changes were observed in mean values of ANP and BNP in uroseptic group I. Similar changes were found in mean values of the three peptides in uroseptic groups II and III.

Table 3 shows the clinical parameters of uroseptic patients depending on the disease severity. There were no significant differences in age, sex distribution, WBC, Scr, and Ccr among the three groups. No significant differences were detected in plasma concentrations of CRP, LAC, and PCT between sepsis and severe sepsis, while a significant difference was observed in plasma ET (*p* < 0.05). However, plasma concentrations of ET, CRP, LAC, and PCT were significantly higher in septic shock than in sepsis (*p* < 0.05).

As shown in Table 4, ET was the most important factor associated with ADM, ANP, and BNP in the uroseptic patients. Stepwise multiple regression analysis of independent parameters (WBC, ET, CRP, LAC, and PCT) related to the values of plasma ADM, ANP, and BNP was also conducted.

Figure 1(d) shows the plasma concentrations of ADM, ANP, and BNP in uroseptic patients depending on the disease severity. The mean value of ADM was significantly higher in septic shock (70.05 ± 21.21 ng/L) than in severe sepsis (55.90 ± 15.31 ng/L) and sepsis (37.75 ± 18.84 ng/L) (*p* < 0.05). There was also a significant difference in mean ADM value between severe sepsis and sepsis (*p* < 0.05). Similar changes were found in mean values of ANP and BNP.

**Figure 2:**
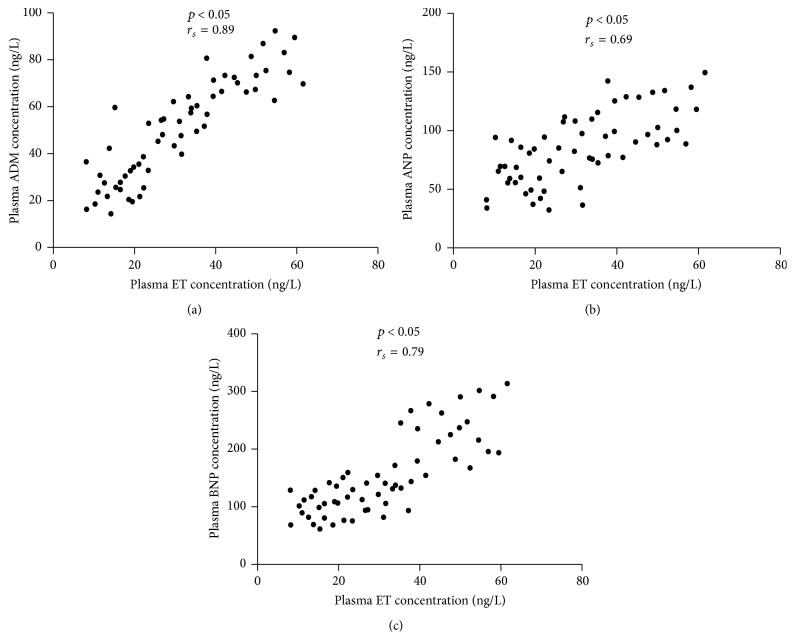
Relationship of plasma ET concentration to values of ADM (a), ANP (b), and BNP (c) in all uroseptic patients.

Scatterplots of Figure 2 show relationship of plasma ET concentration to plasma concentrations of ADM (a), ANP (b), and BNP (c) in uroseptic patients. Plasma ET concentration was positively related to plasma concentrations of ADM, ANP, and BNP.

**Figure 3:**
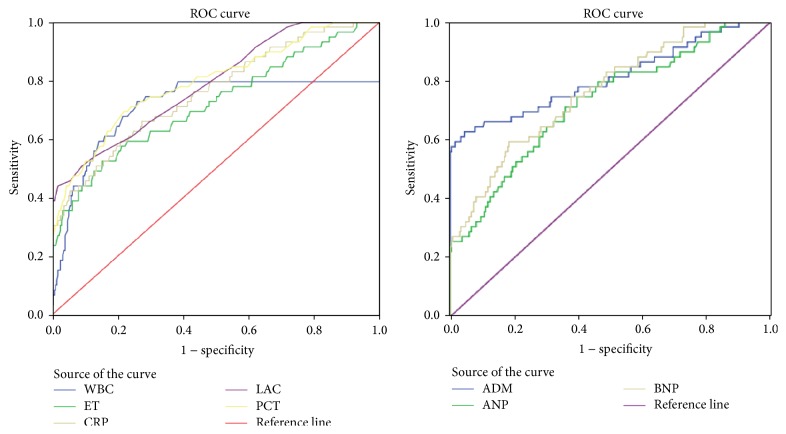
The ROC curves of ET, WBC, CRP, LAC, PCT, ADM, ANP, and BNP for predicting URS-induced urosepsis in UUO patients.

The ROC curves of WBC, ET, CRP, LAC, PCT, ADM, ANP, and BNP for urosepsis are shown in Figure 3. The AUCs are listed in Table 5. The AUC of ADM was 0.811 which was higher than those of WBC (0.712), ET (0.719), CRP (0.758), LAC (0.787), PCT (0.793), ANP (0.728), and BNP (0.764).

**Figure 4:**
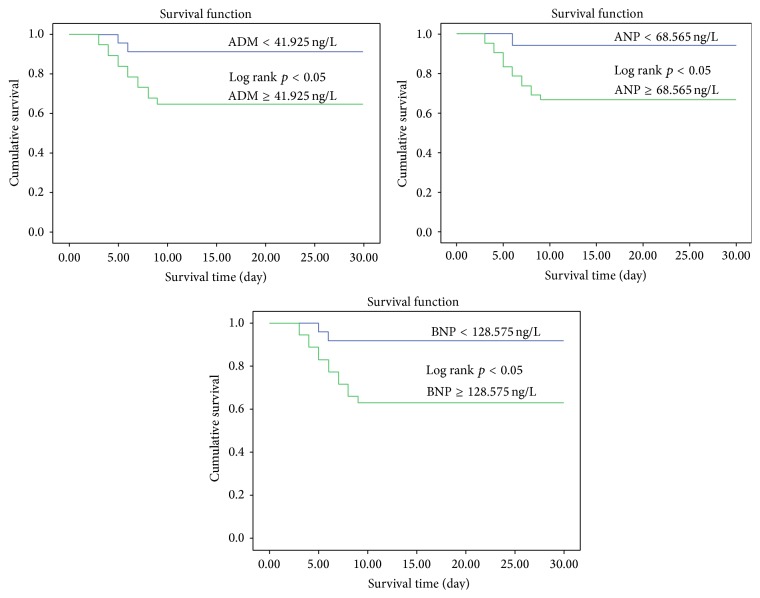
Kaplan-Meier curves for 69 uroseptic patients subdivided into two groups according to the cutoff values of ADM, ANP, and BNP in plasma.

Kaplan-Meier curves for ADM, ANP, and BNP are depicted in Figure 4. The cutoff values of ADM, ANP, and BNP were 41.925, 68.565, and 128.575 ng/L, respectively, for prognosis in uroseptic patients. The survival rates were 64.9%, 66.7%, and 62.9%, respectively, whose values of the three peptides were above the cutoff values, whereas the survival rates were 90.9%, 94.1%, and 91.7%, respectively, whose values of the three peptides were below the cutoff values. There was a significant difference in survival rates between the groups above and below the cutoff values (*p* < 0.05).

## 4. Discussion

Ureteral calculi represent a common condition that urologists encounter in everyday practice. Ureteroscopy is one of the most important treatment options for ureteral calculi that do not pass spontaneously or are unlikely to do so [19]. This procedure carries the risk of postoperative urosepsis affecting UUO patients undergoing ureteroscopy and laser lithotripsy [20]. Urosepsis, uroseptic shock, and the ensuing multiple organ failure continue to be the most common causes of death in critically ill patients of urological department admitted to intensive care unit (ICU) [21]. In urosepsis, as in other types of sepsis, the severity of sepsis depends mostly upon the host response [22]. Human ADM, consisting of 52 amino acids, has a ring structure formed by a disulfide bond and an amidated carboxyl terminus and belongs to a family of calcitonin gene-related peptides [23]. Nowadays, it has been demonstrated that ADM can be synthesized by various other tissues including endothelial and vascular smooth muscle cells, myocardium, and central nervous system [24]. It has multiple functions in a wide range of tissues and acts mainly as a vasodilatory and proliferation-inhibitory factor in cardiovascular system [25]. It was recently reported that ADM plays a central role in initiating the hyperdynamic response during the early stages of sepsis and was a useful predictor for development of severe sepsis and septic shock [10, 26]. The ANP and BNP are similar to ADM in cardiovascular effects including natriuresis, diuresis, and vasodilatation, thereby reducing fluid volume and blood pressure [13, 27]. ANP is a 28-amino-acid peptide chiefly synthesized and released by atrial myocytes in response to atrial distension and stretch, whereas BNP is a 32-amino-acid peptide synthesized and released by ventricular myocytes in response to ventricular stretch or pressure overload [28]. Pro-ANP is a valuable biomarker for prediction of severity of septic patients [29, 30]. The plasma BNP concentrations were increased in patients with severe sepsis or septic shock and poor outcome was associated with high BNP levels [31, 32].

In the current study, the uroseptic rates were significantly higher and higher in proportion to the irrigation pressure. This result was further confirmed by logistic regression analysis of risk factors for urosepsis which showed irrigation pressure was an independent risk factor. Therefore, this result may provide a guide that it is necessary to perform URS under lower irrigation pressure in the clinical practice. Moreover, stone size was another independent risk factor for urosepsis. It can be easily explained that the operation time is longer with bigger stone size.

In our study, plasma levels of ADM, ANP, and BNP were higher in uroseptic patients after URS than before URS and in controls, as well as WBC and plasma concentrations of ET, CRP, LAC, and PCT, but Scr and Ccr remained unchanged. No significant differences were found between uroseptic patients before URS and controls. Moreover, the plasma concentrations of ADM, ANP, and BNP were related to the severity of disease, as well as plasma concentrations of ET, CRP, LAC, and PCT [9, 33, 34]. It can be inferred that WBC and plasma concentrations of ET, CRP, LAC, and PCT had some predictive value for urosepsis and disease severity, in agreement with the literature [35]. ADM, along with ANP and BNP, may participate in initiating the hyperdynamic response during the early stages of sepsis because of their similar physiological functions, in agreement with the literature [16, 26]. Endotoxin was identified as the most important factor in uroseptic patients. This result was confirmed by stepwise multiple regression analysis of independent parameters related to plasma concentrations of ADM, ANP, and BNP. Plasma endotoxin concentration was not only correlated with values of ADM but also related to the values of ANP and BNP in uroseptic patients. We can infer that URS may cause endotoxin absorption which is proportional to the irrigation pressure. The elevated plasma endotoxin concentration may subsequently result in ADM secretion and myocardial cell injury which are responsible for the elevated plasma levels of ADM, ANP, and BNP. Indeed, the mechanism of ADM secretion in large part relates to the effects of lipopolysaccharide (LPS) stimulation which is the most important ingredient of endotoxin. Moreover, it can be inferred that urosepsis secondary to endotoxin absorption can lead to endotoxic cardiomyopathy.

Biomarkers play important roles in diagnosis, differential diagnosis, risk stratification, therapeutic monitoring, and prognosis in sepsis. Many features of pathophysiologic progression correlate with the severity and outcome of the disease and become candidate prognostic biomarkers [36]. Our study showed that the older biomarkers of WBC and plasma concentrations of ET, CRP, LAC, and PCT were predictive indicators of urosepsis according to their AUCs. Our data seem to be compatible with the previous report [35]. The predictive value of PCT is still superior to the other biomarkers. However, the most valuable predictor is ADM and ANP and BNP also have some predictive value in urosepsis according to their AUCs, in agreement with the literature [10, 30, 32].

Several clinical studies have demonstrated that ADM, ANP, and BNP are predictors of adverse outcome in patients with sepsis, but most of these studies were conducted in the ICU and contained relatively small sample sizes. The prognostic value of ADM, ANP, and BNP in uroseptic patients induced by URS in the urological department is still undefined.

Our results suggest that the prognostic value of ADM is superior to ANP and BNP, and ADM, ANP, and BNP are robust independent predictors of in-hospital death in uroseptic patients. In uroseptic patients with ADM, ANP, and BNP levels above the cutoff values of our study, the in-hospital mortality was 35.1%, 33.3%, and 36.1%, respectively, with all patients dying within the first 10 days. In uroseptic patients with ADM, ANP, and BNP levels below the cutoff values, survival rates were 90.9%, 94.1%, and 91.7%, respectively, at the 30-day follow-up. These results demonstrate that ADM, ANP, and BNP are strong predictors of adverse outcome in patients with urosepsis.

## 5. Conclusions

In summary, with the physiological roles of ADM taken together, our study shows that ADM, along with ANP and BNP, may participate in initiating the hyperdynamic response during the early stages of sepsis in uroseptic patients. In addition, ADM, ANP, and BNP are strong predictors of adverse outcome in patients with urosepsis.

## Figures and Tables

**Table 1:** Baseline characteristics of study subjects.

Characteristics	Control (*n* = 90)	UUO (*n* = 332)			*p* value
		Group I (*n* = 118)	Group II (*n* = 132)	Group III (*n* = 82)	
Age (years)	41.3 ± 12.6	43.2 ± 12.1	43.3 ± 11.6	43.6 ± 12.8	>0.05
Gender					
Men	50 (55.6%)	66 (55.9%)	73 (55.3%)	46 (56.1%)	>0.05
Women	40 (44.4%)	52 (44.1%)	59 (44.7%)	36 (43.9%)	
Side					
Left	—	60 (50.8%)	67 (50.8%)	42 (51.2%)	>0.05
Right	—	58 (49.2%)	65 (49.2%)	40 (48.8%)	
Stone site					
Proximal ureter	—	37 (31.4%)	40 (30.3%)	25 (30.5%)	>0.05
Mid ureter	—	41 (34.7%)	45 (34.1%)	28 (34.1%)	
Distal ureter		40 (33.9%)	47 (35.6%)	29 (35.4%)	
Stone size (mm)	—	9.2 ± 4.1	9.6 ± 4.5	10.3 ± 5.2	>0.05
WBC (×10^9^/L)	7.1 ± 1.5	7.2 ± 1.6	6.8 ± 1.5	7.0 ± 1.7	>0.05
ET (ng/L)	3.6 ± 1.4	3.5 ± 1.2	3.6 ± 1.6	3.5 ± 1.7	>0.05
CRP (mg/L)	6.0 ± 2.2	5.8 ± 2.1	5.9 ± 2.1	6.2 ± 2.1	>0.05
LAC (mmol/L)	1.1 ± 0.5	1.2 ± 0.6	1.3 ± 0.6	1.2 ± 0.5	>0.05
PCT (ng/mL)	0.24 ± 0.13	0.23 ± 0.12	0.25 ± 0.13	0.22 ± 0.10	>0.05
Scr (*μ*mol/L)	75 ± 12	77 ± 14	76 ± 13	74 ± 11	>0.05
Ccr (mL/min)	100 ± 10	101 ± 12	99 ± 11	102 ± 14	>0.05

UUO: unilateral ureteral obstruction; WBC: white blood cell count; ET: endotoxin; CRP: C-reactive protein; LAC: lactate; PCT: procalcitonin; Scr: serum creatinine; Ccr: creatinine clearance.

The normal values are as follows: WBC, 4.0~10.0 × 10^9^/L; ET, 0~10 ng/L; CRP, 0~10 mg/L; LAC, 0~2.4 mmol/L; PCT, 0~0.5 ng/mL; Scr, 53~106 *μ*mol/L (male), 44~97 *μ*mol/L (female); Ccr, 80~120 mL/min.

**Table 2:** Parameters of uroseptic patients before and after URS.

Parameters	Uroseptic group I (*n* = 10)		Uroseptic group II (*n* = 24)		Uroseptic group III (*n* = 25)	
	At diagnosis	After URS	At diagnosis	After URS	At diagnosis	After URS
WBC (×10^9^/L)	7.5 ± 1.8	13.4 ± 5.8^*∗*^	6.9 ± 1.6	15.6 ± 6.6^*∗*^	7.2 ± 1.9	17.2 ± 9.4^*∗*^
ET (ng/L)	3.8 ± 1.9	22.1 ± 10.2^*∗*^	3.4 ± 1.8	29.5 ± 14.4^*∗*^	3.7 ± 1.7	36.4 ± 15.6^*∗*^
CRP (mg/L)	5.0 ± 2.4	59.2 ± 19.0^*∗*^	5.2 ± 2.0	109.7 ± 48.9^*∗*^	6.0 ± 2.2	145.9 ± 59.3^*∗*^
LAC (mmol/L)	1.4 ± 0.6	2.4 ± 0.9^*∗*^	1.1 ± 0.6	2.3 ± 0.8^*∗*^	1.1 ± 0.5	2.5 ± 0.9^*∗*^
PCT (ng/mL)	0.20 ± 0.13	0.59 ± 0.23^*∗*^	0.25 ± 0.14	0.58 ± 0.24^*∗*^	0.24 ± 0.11	0.60 ± 0.24^*∗*^
Scr (*μ*mol/L)	73 ± 8	74 ± 10	75 ± 10	77 ± 13	74 ± 9	75 ± 12
Ccr (mL/min)	101 ± 8	100 ± 11	100 ± 9	99 ± 12	99 ± 10	97 ± 14

URS: ureteroscopy; WBC: white blood cell count; ET: endotoxin; CRP: C-reactive protein; LAC: lactate; PCT: procalcitonin; Scr: serum creatinine; Ccr: creatinine clearance.

^*∗*^ *p* < 0.05, compared with subjects at diagnosis.

**Table 3:** Parameters of uroseptic patients depending on the disease severity after URS.

Parameters	Sepsis (*n* = 25)	Severe sepsis (*n* = 24)	Septic shock (*n* = 10)
Age (years)	44.2 ± 12.9	45.8 ± 12.2	45.1 ± 13.5
Gender (male : female)	12 : 13	13 : 11	4 : 6
WBC (×10^9^/L)	13.7 ± 9.6	17.6 ± 6.1	17.5 ± 5.4
ET (ng/L)	21.9 ± 10.0	35.3 ± 15.1^*∗*^	44.4 ± 11.4^*∗*^
CRP (mg/L)	89.5 ± 46.8	116.8 ± 50.9	183.1 ± 51.1^*∗*#^
LAC (mmol/L)	2.1 ± 0.7	2.6 ± 0.8	2.9 ± 0.8^*∗*^
PCT (ng/mL)	0.50 ± 0.23	0.62 ± 0.22	0.74 ± 0.19^*∗*^
Scr (*μ*mol/L)	73 ± 11	76 ± 13	80 ± 15
Ccr (mL/min)	101 ± 8	98 ± 11	95 ± 14

URS: ureteroscopy; WBC: white blood cell count; ET: endotoxin; CRP: C-reactive protein; LAC: lactate; PCT: procalcitonin; Scr: serum creatinine; Ccr: creatinine clearance.

^*∗*^ *p* < 0.05, compared with sepsis; ^#^ *p* < 0.05, compared with severe sepsis.

**Table 4:** Stepwise multiple regression analysis of significant factors for ADM, ANP, and BNP in uroseptic patients.

Variables	ADM (ng/L)			ANP (ng/L)			BNP (ng/L)		
	*B*	*t*	*p*	*B*	*t*	*p*	*B*	*t*	*p*
WBC (×10^9^/L)	0.020	0.106	0.916	−0.297	−0.694	0.491	−0.567	−0.689	0.494
ET (ng/L)	0.847	4.835	0.000	0.979	2.445	0.018	3.797	4.947	0.000
CRP (mg/L)	0.034	1.058	0.295	0.100	1.367	0.177	−0.120	−0.858	0.395
LAC (mmol/L)	4.404	1.964	0.055	7.101	1.386	0.172	12.994	1.323	0.192
PCT (ng/mL)	13.557	1.550	0.127	−7.611	−0.381	0.705	−16.361	−0.427	0.671

ADM: adrenomedullin; ANP: atrial natriuretic peptide; BNP: brain natriuretic peptide; WBC: white blood cell count; ET: endotoxin; CRP: C-reactive protein; LAC: lactate; PCT: procalcitonin.

**Table 5:** The AUCs of WBC, ET, CRP, LAC, PCT, ADM, ANP, and BNP.

Variables	Area	SE	*p*	95% CI	
				Lower	Upper
WBC (×10^9^/L)	0.712	0.049	0.000	0.615	0.809
ET (ng/L)	0.719	0.041	0.000	0.639	0.799
CRP (mg/L)	0.758	0.037	0.000	0.686	0.830
LAC (mmol/L)	0.787	0.034	0.000	0.720	0.854
PCT (ng/mL)	0.793	0.035	0.000	0.724	0.862
ADM (ng/L)	0.811	0.038	0.000	0.737	0.885
ANP (ng/L)	0.728	0.037	0.000	0.655	0.801
BNP (ng/L)	0.764	0.034	0.000	0.697	0.831

WBC: white blood cell count; ET: endotoxin; CRP: C-reactive protein; LAC: lactate; PCT: procalcitonin; ADM: adrenomedullin; ANP: atrial natriuretic peptide; BNP: brain natriuretic peptide.
